# The *FBXL* Gene Family in Tobacco (*Nicotiana tabacum* L.): Identification and Expression Response to TMV and Abiotic Stresses

**DOI:** 10.3390/antiox15020246

**Published:** 2026-02-13

**Authors:** Jiaxin Li, Jia Shen, Fang Wang, Wei Wang, Yifeng Yan, Xiaolu Pan, Chaoqiang Jiang, Huaying Yang, Qing Dong

**Affiliations:** 1 Institute of Industrial Crops, Anhui Academy of Agricultural Sciences, Hefei 230001, China; lijiaxin@aaas.org.cn (J.L.); shenjia@aaas.org.cn (J.S.); wangwei@aaas.org.cn (W.W.); yanyifeng@aaas.org.cn (Y.Y.); panxiaolu@aaas.org.cn (X.P.); cqjiang@aaas.org.cn (C.J.); 2 Crop Research Institute, Anhui Academy of Agricultural Sciences, Hefei 230031, China; 3 Institute of Plant Protection and Agro-Products Safety, Anhui Academy of Agricultural Sciences, Hefei 230031, China; wangfang1378@aaas.org.cn

**Keywords:** *Nicotiana tabacum* L., FBXL family, Tobacco mosaic virus (TMV), ROS, stress responses

## Abstract

F-box-LRR (FBXL) proteins are crucial components of the SCF ubiquitin ligase complex, regulating diverse processes such as development and stress responses in plants. However, the FBXL family in tobacco (*Nicotiana tabacum* L.) remains poorly characterized. This study performed the first genome-wide analysis of the *FBXL* gene family in tobacco and identified 47 *NtaFBXL* genes. Phylogenetic analysis classified them into five clades, among which Clade III exhibited notable expansion. Promoter analysis revealed abundant stress- and hormone-related cis-elements. Expression profiling demonstrated tissue-specific patterns and strong responses to drought, ABA, IAA, and TMV infection. Importantly, six genes exhibited a significant negative correlation with TMV accumulation, suggesting their potential roles in antiviral defense. Moreover, both drought and TMV stress triggered a disturbance of redox homeostasis, a dynamic process that was closely associated with the expression of specific *NtaFBXL* genes, characterized by upregulated antioxidant enzymes (SOD, POD, CAT) and accumulated oxidative markers (H_2_O_2_, MDA). Collectively, this study provided a foundational resource for understanding the function of *NtaFBXLs* and identified key candidate genes for the genetic improvement of stress resistance in tobacco.

## 1. Introduction

In eukaryotic cells, protein degradation is tightly and elaborately regulated. Among the key regulators, the SCF (Skp1-Cullin-F-box) complex acts as a critical multi-subunit E3 ubiquitin ligase within the ubiquitin–proteasome system [1]. This complex specifically identifies target proteins and facilitates their ubiquitination, thereby marking them for subsequent degradation by the 26S proteasome [2,3]. Within the SCF complex, F-box proteins function as the primary determinants of substrate specificity [4,5,6]. The F-box protein family derives its name from the conserved F-box domain and exhibits considerable size and functional diversity in plants [7]. For example, genome-wide studies have revealed approximately 692 F-box genes in *Arabidopsis thaliana*, 779 in *Oryza sativa* (rice), 509 in *Glycine max* (soybean), 359 in *Zea mays* (maize), and 409 in *Triticum aestivum* (wheat) [8,9,10,11]. F-box proteins typically exhibit a modular structure: a highly conserved N-terminal F-box domain (approximately 40–60 amino acids) interacts with the Skp1 component of the SCF core complex, thereby anchoring the F-box protein to the ligase; conversely, the highly variable C-terminal domain determines substrate specificity [12,13,14]. Based on the different C-terminal domains, such as Leucine-Rich Repeat (LRR), Kelch domains, and WD40 domains, F-box proteins are classified into multiple subfamilies [15,16,17].

The LRR domain is a key mediator of protein–protein interactions and plays a crucial role in plant immunity and development [18,19]. Notably, LRR-containing proteins such as the LRR receptor kinases (LRR-RKs) and NBS-LRR disease resistance proteins activate defense responses by recognizing pathogen-associated molecular patterns (PAMPs) or pathogen effectors [20,21,22,23]. A well-documented example is the *N* gene in tobacco, an NBS-LRR protein, which confers specific resistance to Tobacco mosaic virus (TMV) through its LRR domain-mediated recognition of the TMV effector [24,25,26]. Moreover, LRR-RKs are widely involved in perceiving peptide hormones and regulating plant growth and developmental processes [27,28,29].

As a significant subgroup of the F-box protein family, the F-box-LRR (FBXL) subfamily has been systematically identified across various plant species, including Arabidopsis (59), rice (65), soybean (45), cotton (121), and tea plant (37) [8,30,31,32]. Accumulating evidence indicates that FBXL proteins respond to hormonal and developmental signals through the ubiquitination and subsequent degradation of specific target substrates. For example, in Arabidopsis, the FBXL protein TIR1 serves as an auxin receptor, driving Aux/IAA degradation and auxin-responsive transcriptional regulation [33]. Another FBXL protein, ORE9, restricts leaf longevity by mediating the degradation of proteins involved in delaying the senescence process [34]. In wheat, *TaFBXL* regulates the TaGPI-AP protein level in response to exogenous auxin treatment [35]. Moreover, overexpression of *GmFBXL12* in soybean significantly influences seed size, underscoring the potential significance of *FBXL* genes in yield-related traits [30].

Cultivated tobacco (*Nicotiana tabacum* L.) is an important economic crop that is widely used in agricultural production and industrial sectors, especially in the fields of tobacco products and biopharmaceuticals. Its genome originates from the hybridization of *Nicotiana tomentosiformis* and *Nicotiana sylvestris*. This complex genomic architecture has complicated genetic studies and resulted in less comprehensive functional gene annotation compared to model plants like Arabidopsis and rice. Consequently, research on specific gene families, including the *FBXL* family, has progressed more slowly in tobacco. The recently released NtaSR1 v1.0 genome assembly overcomes previous limitations with unprecedented quality: it achieves a contig N50 of 1.2 Mb, a scaffold N50 of 58 Mb, and a functional annotation completeness exceeding 92% (with 95% of conserved orthologs identified via BUSCO analysis) [36]. These metrics represent a significant improvement over the previously widely used tobacco reference genome (Nitab4.5) and provide a robust foundation for reliable genome-wide family identification in this study. *FBXL* genes have been studied in Arabidopsis, rice, soybean, but not yet systematically in tobacco, where their role in stress responses is unknown. We hypothesized that the *NtaFBXL* gene family has undergone expansion and functional diversification in tobacco, with specific members playing crucial roles in the plant’s adaptation to both abiotic and biotic stresses. To test this hypothesis, the present study aimed to: (1) perform a genome-wide identification and phylogenetic analysis of all *NtaFBXL* genes; (2) characterize their gene structures, conserved motifs, and promoter cis-elements; (3) profile their expression patterns and their dynamic responses to stress and exogenous hormones. This study delivers a foundational resource for dissecting the functional roles of the *NtaFBXL* gene family in tobacco, while also furnishing valuable insights into plant ubiquitin-mediated stress regulation and identifying candidate genetic targets. These outputs can directly inform future genetic engineering strategies to enhance both abiotic stress resistance and biotic stress tolerance in tobacco.

## 2. Materials and Methods

### 2.1. Identification and Characterization of FBXL Members in Nicotiana tabacum

Genomic data for *Nicotiana tabacum* cv. Petite Havana SR1 (assembly version: NtaSR1 v1.0) were obtained from the Nicomics database (http://lifenglab.hzau.edu.cn/Nicomics, accessed on 16 April 2024) [36]. A Hidden Markov Model (HMM) search was performed with the HMM files of the F-box domain (PF00646), retrieved from the Pfam database as a query (E-value < 1 × 10^−5^) [37]. Candidate genes were further validated for domain architecture using CDD and SMART, and those encoding proteins containing both an N-terminal F-box domain and C-terminal LRR domain(s) were definitively classified as *FBXL* family members [38,39]. Physicochemical properties of NtaFBXL proteins were predicted using TBtools(v2.056), and subcellular localizations were inferred with WoLF PSORT. (https://www.genscript.com/wolf-psort.html, accessed on 18 October 2024).

### 2.2. Multiple Sequence Alignment and Phylogenetic Analysis

The full-length sequences of *NtaFBXLs* were determined by selecting the longest transcript. To elucidate the phylogenetic relationships among FBXL homologs from multiple species, FBXL protein sequences from *Arabidopsis thaliana* (59 members) and *Oryza sativa* (65 members) were included in the analysis [8]. Multiple sequence alignment of the FBXL proteins was performed using ClustalW, and a phylogenetic tree was constructed using MEGA11 (v11.0.13) with the maximum likelihood (ML) method, the WAG evolutionary model, and 1000 bootstrap replicates [40]. Phylogenetic trees were visualized using the ITOL online tool (http://itol.embl.de/, accessed on 7 Novermber 2024) [41].

### 2.3. Gene Structure and Conserved Motif Analysis of NtaFBXL Members

Conserved motifs were identified using the MEME suite with the following parameters: maximum number of motifs = 10, and optimum motif width between 6 and 50 amino acids (http://meme-suite.org/, accessed on 8 Novermber 2024) [42]. Structural information of the *NtaFBXL* genes, including exons and introns, was obtained in Generic File Format (GFF3) from the Nicomics database. The batch SMART function in the TBtools was utilized to detect conserved domains within the *NtaFBXL* proteins [43].

### 2.4. Chromosome Localization and Collinearity Analysis

The chromosomal location information of *NtaFBXL* genes, along with chromosome length and gene density information, was obtained from the tobacco genome database. Subsequently, the precise physical positions of these genes on the chromosomes were mapped using TBtools. Gene duplication events and synteny blocks among *FBXL* genes from *Nicotiana tabacum*, *Arabidopsis thaliana* and *Oryza sativa* were analyzed using the TBtools “One Step MCScanX” function with the following parameters: E-value ≤ 1 × 10^−10^ and retention of the top 5 blast hits per gene. The collinear pairs associated with the *FBXL* family were extracted, and a collinearity diagram was constructed using Circos (https://circos.ca/) and TBtools software. The genomic information for Arabidopsis and rice was sourced from the Ensembl Plants database (https://plants.ensembl.org/, accessed on 12 October 2024).

### 2.5. Prediction of Cis-Elements in NtaFBXLs

For each of the 47 *NtaFBXL* genes, a 2000 bp region upstream of the ATG start codon was extracted from the tobacco genome. These sequences were subsequently analyzed to identify potential cis-regulatory elements using the PlantCARE database (http://bioinformatics.psb.ugent.be/webtools/plantcare/html/, accessed on 22 October 2024) [44]. Functional motifs associated with light signaling, hormone response, environmental stress, and developmental processes were selected and reported.

### 2.6. Gene Ontology and KEGG Analysis

Protein sequences of NtaSR1 were annotated using eggNOG-mapper (http://eggnog-mapper.embl.de, accessed on 29 July 2024). Subsequently, Gene Ontology and KEGG pathway enrichment analyses were performed using TBtools. Circular graphs and bar graphs were used to visually display the significantly enriched GO terms in the three main categories (BP: Biological Process, CC: Cellular Component, MF: Molecular Function). Significantly enriched KEGG pathways were visualized using bubble plots, where bubble size is proportional to the number of genes and color indicates the level of enrichment significance. *p*-values were adjusted for multiple testing using the Benjamini–Hochberg method, and terms with an adjusted *p*-value (FDR) < 0.05 were deemed significant.

### 2.7. Expression Pattern of Different Tissues

Transcriptome data (RNA-seq) derived from tobacco roots, stems, leaves, and flowers were obtained from the NCBI Sequence Read Archive (SRA) database (Accession: PRJNA208209), which included 3 biological replicates per tissue [45]. The raw sequencing reads were first subjected to quality control and adapter trimming using fastp (v0.23.2) to obtain high-quality clean data. The cleaned reads were then aligned to the NtaSR1 reference genome using HISAT2 (v2.2.1). Read counts for each gene were generated from the alignment files using featureCounts from the Subread package (v2.0.0), with gene annotation from the reference GFF file. Finally, gene expression levels were normalized to Transcripts Per Million (TPM) using the DESeq2 package (v1.40.0) in R.

### 2.8. Plant Materials Quantitative Real-Time PCR Analysis

Tobacco (*Nicotiana tabacum* L. cv. SR1) seeds were surface-sterilized and sown in a 128-cell seedling tray containing sterilized mixed soil (vermiculite:humus = 1:1, *v*/*v*). Plants were grown in a controlled-environment chamber under a 16 h light/8 h dark photoperiod at 25 °C and 60% relative humidity. To ensure precise and uniform application of chemical stimuli, seedlings designated for drought stress (simulated by 10% (*w*/*v*) PEG6000), abscisic acid (ABA, 100 μM), and indole-3-acetic acid (IAA, 100 μM) treatments were gently transferred to a hydroponic system containing half-strength Hoagland’s nutrient solution. Seedlings for Tobacco mosaic virus (TMV) inoculation were transplanted into individual pots containing the same soil mixture to maintain a natural growth context for pathogen-host interaction studies. All transplanted plants underwent a 7-day acclimation period under the same controlled conditions to recover from transplanting stress. After acclimation, stress treatments were applied. For hydroponically grown plants, stressors were added directly to the nutrient solution. For soil-grown plants, TMV inoculation was performed by dusting the second fully expanded leaf with carborundum and rubbing with TMV inoculum (20 μg/mL in phosphate buffer). Leaf samples were collected at the following time points: 0, 1, 3, 6, and 12 h post-treatment for drought, ABA, and IAA treatments; and 1, 3, 5, and 7 days post-inoculation for TMV treatment. Three independent biological replicates (individual plants) were used per treatment per time point. All samples were immediately frozen in liquid nitrogen and stored at −80 °C for subsequent analysis.

Total RNA was extracted from the collected samples using the RNAprep Pure Plant Plus Kit (TIANGEN, Beijing, China) following the manufacturer’s protocol. First-strand cDNA synthesis was carried out using ReverTra Ace^®^ quantitative PCR (qPCR) RT Master Mix with gDNA Remover (TOYOBO, Osaka, Japan) to eliminate genomic DNA contamination. qPCR primers were designed using Primer Premier 5.0 and their specificity was verified using TBtools software. qPCR reactions were conducted using THUNDERBIRD SYBR qPCR Mix (TOYOBO, Osaka, Japan) on a CFX96™ Real-Time PCR Detection System (BIO-RAD, Hercules, CA, USA). The relative expression levels of target genes were calculated using the 2^−∆∆CT^ method, with tobacco β-actin gene serving as the internal reference. Each sample was analyzed with three biological replicates, and each biological replicate included three technical replicates to ensure experimental accuracy and reproducibility. Selection of *NtaFBXL* genes for qPCR validation was based on phylogenetic diversity, homology to functionally characterized *FBXLs*, and non-redundancy among collinear gene pairs. All the gene information is detailed in Table S1.

### 2.9. Determination of Physiological Indicators

Physiological indicators were quantified using specified commercial assay kits (Suzhou Geruisi Biotechnology Co., Ltd., Suzhou, China) according to the manufacturer’s instructions. The following kits were employed: superoxide dismutase (SOD, Cat# G0101W, WST-8 method), catalase (CAT, Cat# G0105W, visible colorimetric method), peroxidase (POD, Cat# G0107W, guaiacol oxidation method), malondialdehyde (MDA, Cat# G0109W, thiobarbituric acid method), hydrogen peroxide (H_2_O_2_, Cat# G0168W, chromogenic method), and soluble protein content (Cat# G0417W, Coomassie Brilliant Blue G-250 method) [46].

For sample preparation, frozen leaf tissue (0.1 g) was homogenized in liquid nitrogen and extracted with 1.0 mL of the corresponding kit’s extraction buffer. The homogenate was centrifuged at 12,000× *g* for 15 min at 4 °C, and the resulting supernatant was used for subsequent assays.

SOD activity was determined based on the inhibition of superoxide anion-mediated reduction of WST-8 to a water-soluble formazan dye, measured at 450 nm. One unit (U) of SOD activity was defined as the amount of enzyme required to achieve 50% inhibition under the assay conditions. CAT activity was measured by monitoring the decomposition of H_2_O_2_ at 510 nm via a chromogenic reaction. POD activity was assessed by measuring the oxidation of guaiacol at 470 nm, with one unit defined as an increase of 0.01 in absorbance per minute. MDA content was evaluated by the thiobarbituric acid reaction, measuring absorbance at 532 nm with a correction at 600 nm to eliminate nonspecific turbidity. H_2_O_2_ content was quantified at 510 nm based on the peroxidase-mediated oxidation of a specific chromogen. Soluble protein concentration in the crude extracts was determined using the Coomassie Brilliant Blue G-250 method, with absorbance measured at 600 nm.

All enzyme activities (SOD, CAT, POD) and metabolite contents (MDA, H_2_O_2_) were normalized to the soluble protein content. Each measurement was performed with three independent biological replicates, each consisting of three technical replicates to ensure reproducibility.

### 2.10. Statistical Analysis

All data from qPCR and physiological assays are presented as the mean ± standard deviation (SD) of three independent biological replicates. A biological replicate was defined as a sample derived from an independently grown and treated individual plant, while a technical replicate refers to multiple measurements (e.g., three qPCR reactions) performed on the same biological sample. The bar graphs for qPCR results and enzyme activity data were generated, and statistical analyses were performed using GraphPad Prism (version 10.5). For comparisons across multiple groups (e.g., different time points under a specific stress), a one-way analysis of variance (ANOVA) was employed after verifying the assumptions of normality and homogeneity of variances. Where the ANOVA indicated a significant effect (*p* < 0.05), Tukey’s Honest Significant Difference (HSD) post hoc test was applied for all pairwise comparisons. The correlation analysis between the expression levels of *NtaFBXL* genes and the accumulation of TMV CP1 was assessed by calculating the Pearson correlation coefficient using R software (version 4.3.1). Differences were considered statistically significant at a *p*-value < 0.05.

## 3. Results

### 3.1. Identification of NtaFBXL Gene Family Members

This study identified a total of 47 *NtaFBXL* genes, which were ultimately confirmed and renamed from NtaFBXL1 to NtaFBXL47 based on their physical location on the chromosomes. The gene characteristics are listed in Table S2. Protein lengths ranged from 230 (NtaFBXL5, NtaFBXL10) to 669 (NtaFBXL37, NtaFBXL38) amino acids (aa), with an average of 497 aa. Corresponding molecular weights ranged from 25.51 to 73.03 kDa, consistent with the typical size range of F-box proteins in plants. Theoretical pI values varied from 4.50 (NtaFBXL44, NtaFBXL47) to 9.05 (NtaFBXL5), with most proteins exhibiting acidic or neutral isoelectric points, which may affect substrate interactions under different pH conditions. The instability index values ranged from 32.44 to 59.98, with 34 NtaFBXL proteins predicted to be unstable (>40) and 13 predicted to be stable (≤40), which aligns with their potential requirement for rapid turnover through ubiquitination-mediated regulation. Aliphatic indices (81.10–114.76) indicated high thermal stability, while GRAVY values (−0.473 to 0.235) reflected overall hydrophilicity, facilitating protein interactions in aqueous environments. Subcellular localization predictions revealed a broad distribution pattern: 38, 32, and 28 members were predicted to localize to the nucleus, cytoplasm, and chloroplasts, respectively. Specific examples include nuclear-localized NtaFBXL9/10/21, chloroplast-localized NtaFBXL6/32, and mitochondrial-targeted NtaFBXL4/20. Notably, 21 members (e.g., NtaFBXL1) were predicted to localize to multiple compartments, suggesting potential roles in cross-compartmental signaling.

### 3.2. Phylogenetic Analysis of NtaFBXL Gene Family Members

**Figure 1:**
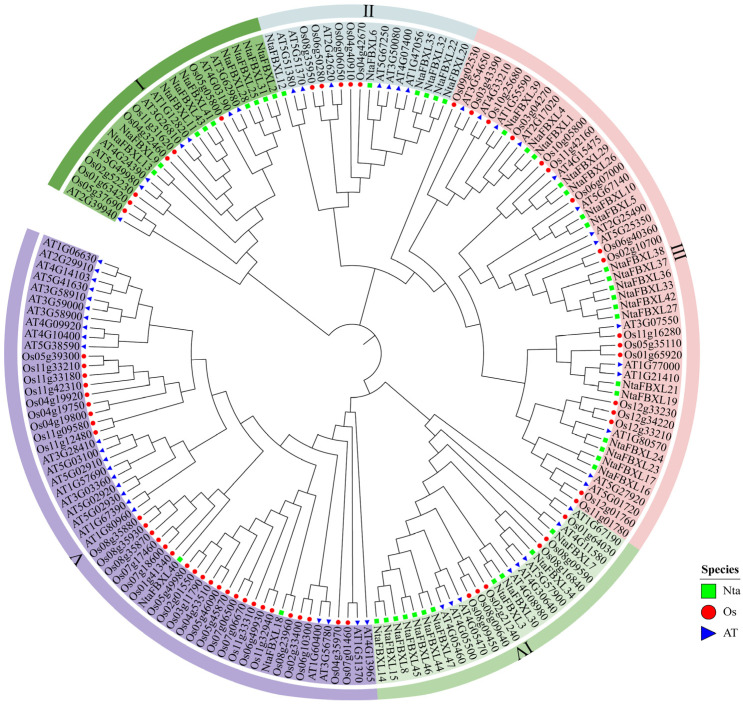
Phylogenetic relationships of *FBXL* genes from *Nicotiana tabacum*, *Oryza sativa*, and *Arabidopsis thaliana*. Green rectangle indicates *NtaFBXLs*, red circle indicates *OsFBXLs*, and blue triangle indicates *AtFBXLs*. *FBXLs* are classified into five major clades (I–V), each marked with a distinct background color. The phylogenetic tree was constructed using the ML method with the WAG evolutionary model and 1000 bootstrap replicates.

To elucidate the evolutionary relationships among tobacco, Arabidopsis and rice, a comprehensive phylogenetic analysis was performed based on multiple sequence alignment of 47 *NtaFBXLs*, 59 *AtFBXLs*, and 65 *OsFBXLs*. As depicted in the phylogenetic tree (Figure 1), all FBXL proteins were distinctly grouped into five major clades, labeled I through V. These clades represent distinct evolutionary lineages that likely reflect functional divergence or conservation among the FBXL proteins in the three species. Notably, clade III demonstrated the highest concentration of *NtaFBXLs*, suggesting a lineage-specific expansion of this group in tobacco. This pattern may indicate that clade III has undergone rapid gene duplication or retention events in tobacco, potentially contributing to the diversification of biological functions associated with these proteins in this species. Conversely, clade V contained the lowest proportion of *NtaFBXLs* compared to the other clades. This relatively sparse representation may suggest that the functions associated with clade V are either highly conserved across species or are subject to stricter evolutionary constraints in tobacco. Such conservation could point to essential roles that these proteins play in fundamental biological processes shared among the three plant species.

### 3.3. Structural Characteristics of NtaFBXLs

**Figure 2:**
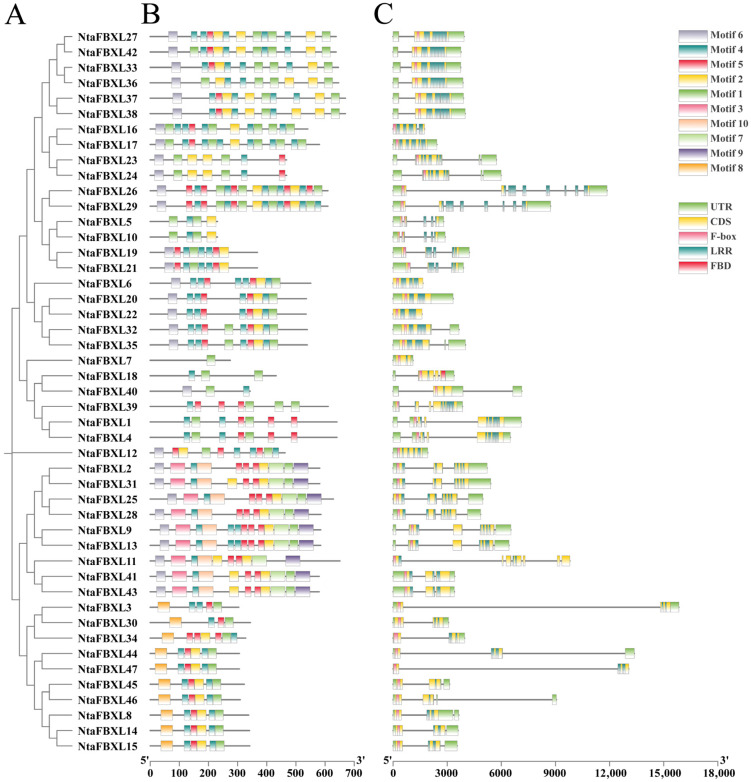
The conserved motifs and gene structures of *NtaFBXLs*. (**A**) Phylogenetics tree of *NtaFBXLs*. (**B**) NtaFBXL proteins Motif prediction. (**C**) Analysis of gene structure of *NtaFBXL* genes.

The phylogenetic tree of the *NtaFBXL* gene family was constructed based on the longest transcript sequences of its 47 members (Figure 2A). Genes clustered within the same branch exhibit similar structural characteristics, indicating close evolutionary relationships. Using the MEME suite, ten conserved motifs were identified (Figure 2B). These motifs are predominantly conserved in both sequence and positional arrangement across most family members, suggesting they represent functionally or structurally important features characteristic of the *NtaFBXL* family. However, the absence or variation of specific motifs in certain genes may reflect functional specialization or divergence. This observed diversity in motif composition among *NtaFBXL* genes likely underlies their involvement in diverse biological processes. All NtaFBXL proteins contain a conserved F-box domain at the N-terminus and one or more LRR domains at the C-terminus (Figure 2C). Analysis of the exon-intron structure within the *NtaFBXL* gene family provides insights into its evolutionary history and potential regulatory mechanisms. Variations in the number, length, and position of introns and exons among members suggest that these structural differences may have contributed to the functional diversification of the gene family during evolution.

### 3.4. Chromosomal Localization and Collinearity Analysis of NtaFBXL Genes

**Figure 3:**
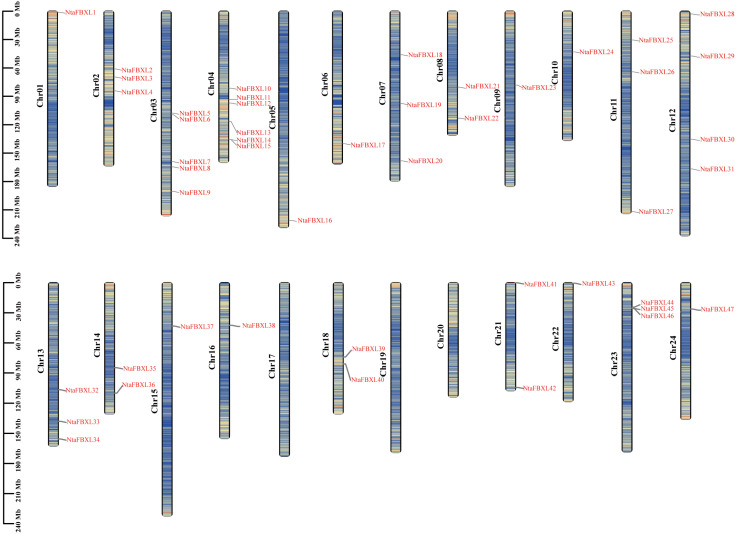
The distribution of *NtaFBXL* genes on chromosomes. Each vertical bar represents a chromosome of *Nicotiana tabacum* L., labeled as Chr01 to Chr24. The position of each *NtaFBXL* gene is marked on the corresponding chromosome, with gene names indicated in red. The scale on the left side of each chromosome denotes length in megabases (Mb). The color scale encodes gene density, ranging from low density (blue) to high density (red).

**Figure 4:**
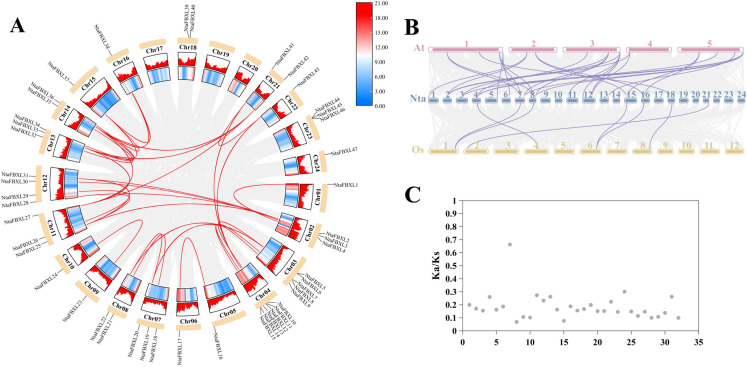
The collinearity relationship of *FBXL* genes. (**A**) The contributory relationship of *NtaFBXLs*. Each outer arc represents a tobacco chromosome, with *NtaFBXL* gene names labeled. The heatmap on chromosome arcs shows gene density. Red lines connect collinear *NtaFBXL* gene pairs; gray lines show collinearity for all genes (background). (**B**) The collinearity relationship among *NtaFBXLs*, *OsFBXLs* and *AtFBXLs*. Chromosomes are shown as bars (numbered). Lines link orthologous *FBXL* gene pairs across species. (**C**) The distribution of Ka/Ks ratio of *NtaFBXLs*. Each dot represents a collinear pair.

Chromosomal localization analysis revealed that the identified *NtaFBXL* genes are widely distributed across 21 of tobacco’s 24 chromosomes, exhibiting distinct distribution patterns (Figure 3). Chromosome 4 harbors the highest number (6 *NtaFBXLs*). Intra-genomic collinearity analysis identified 32 collinear gene pairs among 28 *NtaFBXLs* (Figure 4A), suggesting that this gene family underwent complex gene duplication and rearrangement events during tobacco genome evolution. Inter-genomic collinearity analysis between tobacco and Arabidopsis/rice showed that 15 *NtaFBXLs* share synteny with 21 *AtFBXLs*, while 7 *NtaFBXLs* are syntenic with 5 *OsFBXLs* (Figure 4B). These relationships indicate partial conservation of gene arrangement and functional associations among *FBXL* families in these species post-divergence. Ka/Ks analysis of duplicated *NtaFBXL* pairs revealed all ratios < 1 (range: 0.07–0.66, mean: 0.18; Figure 4C, Table S4), consistent with purifying selection during evolution.

### 3.5. Promoter Cis-Elements Analysis of NtaFBXL Genes

**Figure 5:**
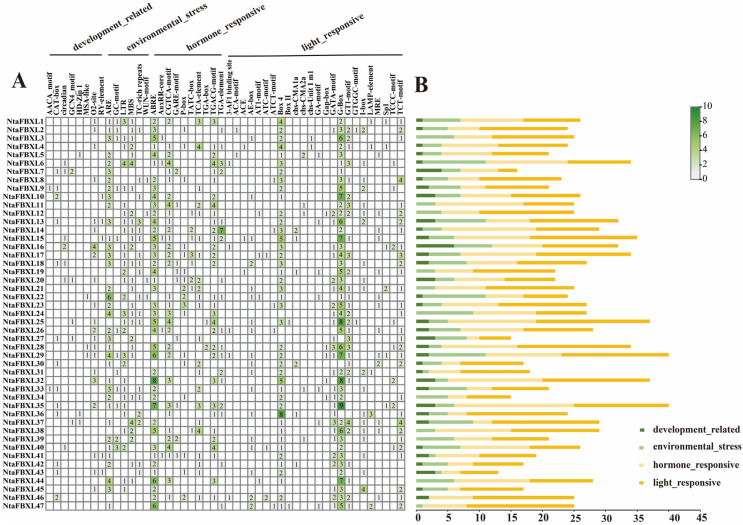
Cis-acting elements within the *NtaFBXL* gene family were identified. (**A**) The number of these elements in the promoter regions of the *NtaFBXL* genes is represented by varying color intensities and numerical values within the grid. (**B**) The different colored histogram represented the sum of the cis-acting elements in each category.

To elucidate the regulatory mechanisms underlying *NtaFBXL* gene expression, cis-acting regulatory elements were classified into four major categories: development-related, environmental stress-responsive, hormone-responsive, and light-responsive elements (Figure 5). Key developmental regulatory motifs identified included the CAT-box and CCAAT-box, known to regulate meristem maintenance, embryonic development, and organogenesis. For environmental stress adaptation, distinct cis-elements associated with abiotic and biotic stress responses were detected. Abiotic stress-related elements encompassed the anaerobic response element (ARE), implicated in hypoxia tolerance and detected in 36 of the 47 *NtaFBXL* genes (76.6%), and the MYB recognition element (MBS), involved in drought response and osmotic adjustment, which was present in 27 genes (57.4%). Biotic stress-related TC-rich repeats—known regulators of pathogen defense gene transcription—were also identified. Hormone-responsive elements exhibited significant enrichment across the *NtaFBXL* family: ABA-responsive elements (ABREs) were identified in 41 of the 47 *NtaFBXL* genes (87.2%), while MeJA-responsive elements were present in 31 genes (66.0%). Subsets of *NtaFBXL* genes contained cis-elements responsive to auxin, salicylic acid, and gibberellin, suggesting roles in hormone-mediated signaling. Light-responsive motifs, including G-boxes associated with photoregulated expression, were also prevalent. Collectively, these findings indicate that *NtaFBXL* genes are subject to complex transcriptional regulation by diverse developmental, hormonal, and environmental signals.

### 3.6. Gene Ontology and KEGG Analysis of NtaFBXL Genes

**Figure 6:**
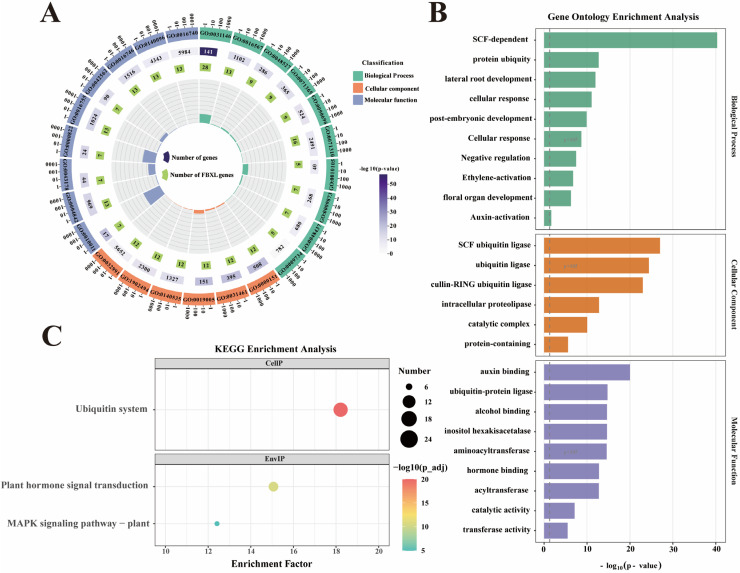
GO and KEGG enrichment analysis of *NtaFBXL* genes. (**A**) Circos plot showing hierarchical distribution of GO terms across three categories for *NtaFBXL* genes. The color gradient corresponds to −log_10_(*p*-value), where higher values indicate stronger statistical significance of GO term enrichment. (**B**) Histogram of top enriched GO terms ranked by significance for *NtaFBXL* genes. The x-axis represents −log_10_(*p*-value). (**C**) Scatter plot of KEGG pathway enrichment results for *NtaFBXL* genes. The color gradient of dots corresponds to −log_10_(*p*-value); dot size represents the number of *NtaFBXL* genes in each pathway, and the x-axis shows the enrichment factor.

To dissect the biological functions of *NtaFBXL* genes, GO and KEGG pathway enrichment analyses were conducted (Figure 6). These analyses delineated molecular mechanisms and biological processes involving *NtaFBXL* genes, providing systems-level insights into their functional roles in tobacco. GO analysis revealed significant enrichment across all three categories: Biological Process, Cellular Component, and Molecular Function. In Biological Process, the most enriched term was “SCF-dependent protein ubiquitylation”, consistent with F-box proteins functioning as substrate-recognition subunits of SCF E3 ubiquitin ligases. This implicates *NtaFBXL* genes in ubiquitin-mediated proteolysis, critical for protein turnover and cellular homeostasis. Enriched terms like “lateral root development” (*p* = 1.09 × 10^−12^) and “cellular response to auxin stimulus” (*p* = 8.45 × 10^−12^) further suggest roles in developmental and hormonal networks regulating root architecture and environmental responses. For Molecular Function, top terms included “auxin binding” (*p* = 1.00 × 10^−20^) and “ubiquitin–protein ligase” (*p* = 1.67 × 10^−15^), indicating potential dual roles in hormone perception and targeted ubiquitination. Complementing the GO analysis, KEGG pathway enrichment provided additional insights into the broader biological contexts in which *NtaFBXL* genes operate. The “ubiquitin system” pathway was among the most significantly enriched, reinforcing the central role of these genes in protein homeostasis through ubiquitin-mediated degradation. Additionally, the identification of the “MAPK signaling pathway” (*p* = 1.19 × 10^−5^) as a significantly enriched pathway implicates *NtaFBXL* proteins in stress response mechanisms and developmental signaling cascades. MAPK pathways are known to mediate responses to various biotic and abiotic stresses, as well as to regulate cell division and differentiation.

### 3.7. Expression of NtaFBXL Family Genes in Different Tissues

**Figure 7:**
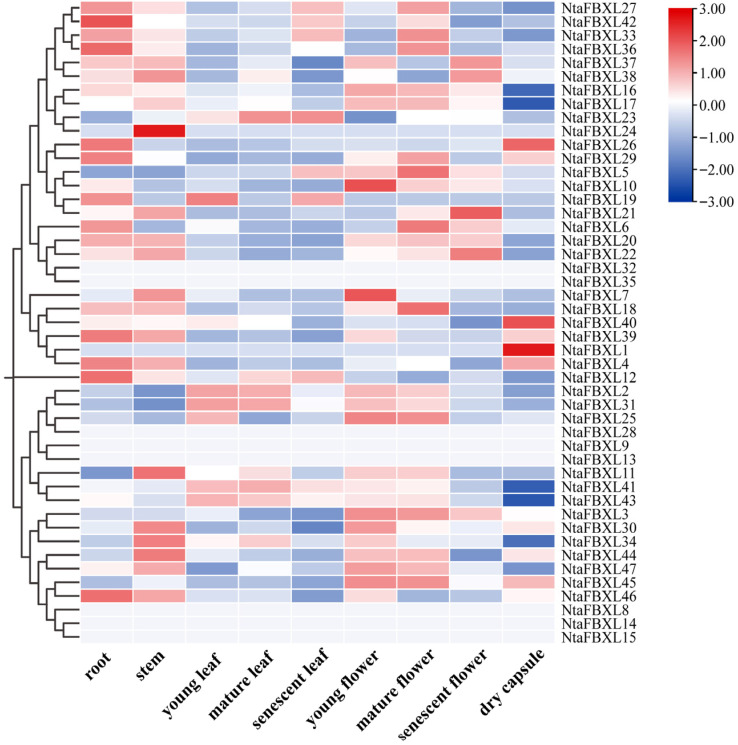
Tissue-specific expression profiles of *NtaFBXL* genes in tobacco revealed by RNA-seq. The color scale at the right indicates expression levels (red: high expression; blue: low expression).

To characterize the tissue-specific functions of *NtaFBXL* genes in tobacco, expression patterns across nine distinct tissues were systematically analyzed using RNA-seq data (Figure 7). The expression heatmap revealed distinct tissue-specific patterns, with elevated transcript abundance for most *NtaFBXL* genes observed in roots, stems, and floral tissues. This spatial expression profile aligns with the results of our GO enrichment analysis, which identified significant associations with terms including “root development” and “floral organ morphogenesis”. The consistent patterns emerging from both expression profiling and functional annotation analyses suggest that *NtaFBXL* genes may have specialized roles in these particular organs.

### 3.8. Expression Pattern of the NtaFBXL Genes Under Various Treatments

**Figure 8:**
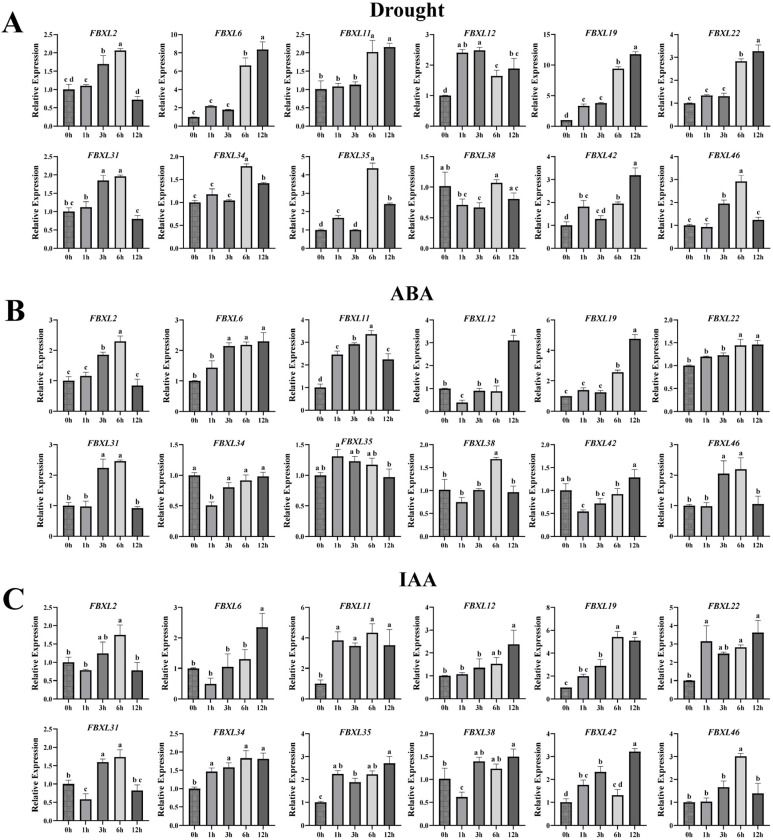
qPCR analysis of *NtaFBXL* gene expression in response to abiotic stress and hormone treatments. (**A**) Drought stress; (**B**) ABA treatment; (**C**) IAA treatment. Expression levels are presented as mean ± SD. Different lowercase letters above bars indicate significant differences among time points (*p* < 0.05) as determined by one-way ANOVA followed by Tukey’s HSD test.

**Figure 9:**
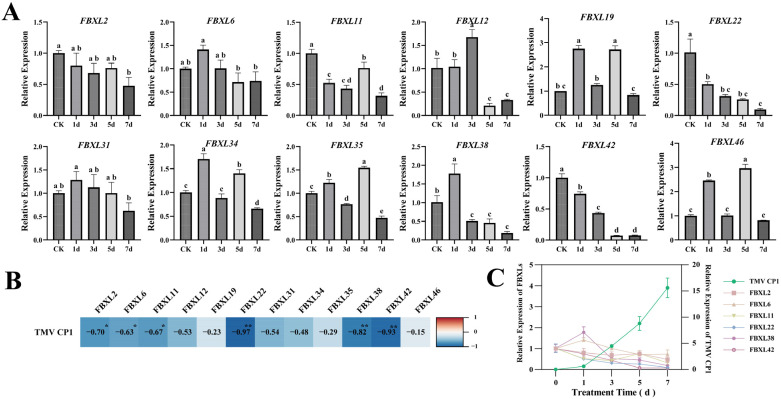
Temporal expression dynamics and correlation analysis of *NtaFBXL* genes in response to TMV inoculation. (**A**) Temporal expression profiles of *NtaFBXL* genes after TMV inoculation detected by qPCR. Data are presented as mean ± SD (*n* = 3), different lowercase letters indicate significant differences among time points (*p* < 0.05) determined by one-way ANOVA followed by Tukey’s HSD test. (**B**) Correlation heatmap between *NtaFBXL* gene expression and TMVCP1 transcript levels. The color gradient represents the Pearson correlation coefficient (*r*). Blue indicates negative correlation, and red indicates positive correlation. ** *p* < 0.01 and * *p* < 0.05. (**C**) Expression dynamics of TMV *CP1* and its significantly negatively correlated genes.

Given the presence of biotic/abiotic stress- and hormone-responsive *cis*-acting elements in *NtaFBXLs* promoters, coupled with enrichment of hormone signal transduction and MAPK pathways in GO/KEGG analyses, we performed qPCR to analyze temporal expression dynamics of 12 selected *NtaFBXL* genes under drought stress, ABA treatment, IAA (auxin) treatment, and TMV inoculation (Figure 8 and Figure 9).

Under drought stress conditions, most *NtaFBXL* genes displayed a temporally regulated upregulation (Figure 8A). The expression of *NtaFBXL12* and *NtaFBXL19* was significantly induced at 1 hpt, with increases of >2.4-fold and >3.0-fold relative to the control, respectively, indicating an early response to water-deficit conditions. In contrast, *NtaFBXL2*, *NtaFBXL31*, and *NtaFBXL46* showed a delayed response, with significant upregulation (approximately 1.70- to 1.95-fold, *p* < 0.05) beginning at 3 h post-treatment (hpt), reaching peak expression levels at 6 hpt, and gradually declining thereafter. Additionally, *NtaFBXL6*, *NtaFBXL11*, and *NtaFBXL22* displayed significant induction at 6 hpt (approximately 2.0- to 6.6-fold, *p* < 0.05), implying a potential role in later stages of the drought response. Expression patterns in response to ABA treatment partially overlapped with those under drought stress. For instance, *NtaFBXL2*, *NtaFBXL19*, and *NtaFBXL31* showed significant upregulation in response to ABA, with expression trends that closely mirrored those seen during drought stress. This similarity supports the well-established role of ABA as a key signaling molecule in drought responses and suggests that these genes may be part of the ABA-mediated signaling pathway. However, distinct regulatory patterns were also observed, as exemplified by *NtaFBXL11*, which exhibited rapid upregulation (2.46-fold, *p* < 0.05) within 1 h of ABA treatment (Figure 8B). In response to IAA treatment, a major auxin involved in plant growth and development, several *NtaFBXL* genes were immediately upregulated (Figure 8C). Specifically, *NtaFBXL11*, *NtaFBXL22*, and *NtaFBXL35* showed rapid induction (reaching 2.24- to 3.08-fold at 1 hpt, *p* < 0.05), suggesting a possible role in auxin signaling pathways that regulate growth-related processes. Interestingly, *NtaFBXL31* and *NtaFBXL38* exhibited a biphasic expression pattern in response to IAA treatment, characterized by initial significant downregulation followed by subsequent upregulation. Collectively, these results indicate that the majority of *NtaFBXL* genes are responsive to drought stress, ABA, and IAA treatments, highlighting their potential involvement in both abiotic stress adaptation and hormone signaling pathways. However, the observed differences in expression kinetics and response patterns among individual genes within the family suggest that they may be regulated through distinct molecular mechanisms. These variations could contribute to the functional diversity of the *NtaFBXL* gene family, enabling it to participate in a wide range of physiological and developmental responses to environmental challenges.

The temporal expression patterns of *NtaFBXL* genes following TMV inoculation revealed distinct and diverse response profiles (Figure 9A). *NtaFBXL2*, *NtaFBXL22*, and *NtaFBXL42* exhibited a consistent trend of sustained downregulation throughout the entire infection time course, suggesting that these genes may be negatively regulated during viral infection and potentially play a role in suppressing viral replication or modulating host defense responses. In contrast, the remaining members of the gene family exhibited more complex expression dynamics. Some genes showed an initial phase of upregulation followed by downregulation, or oscillatory expression patterns characterized by alternating phases of upregulation and downregulation. To further characterize the functional relevance of these expression changes in the context of TMV infection, we detected the expression levels of the TMV coat protein (CP1) at multiple time points post-inoculation using qPCR. CP1 is a key structural component of the virus, and its expression level serves as a reliable indicator of viral replication and accumulation within host cells. By correlating the expression profiles of *NtaFBXL* genes with TMV *CP1* levels, we aimed to uncover potential functional associations between host gene expression and viral propagation. A novel finding from the correlation analysis was that the expression levels of *NtaFBXL2/6/11* were significantly negatively correlated with TMV CP1 accumulation (*p* < 0.05; r = −0.63 to −0.70), while *NtaFBXL22/38/42* showed highly significant negative correlations (*p* < 0.01; r = −0.82 to −0.97) (Figure 9B,C). Given that TMV accumulation increased over time alongside the downregulation of these *NtaFBXL* genes, this negative correlation suggests that reduced expression of these six *NtaFBXL* genes may be associated with enhanced viral accumulation, implying they could play a role in restricting TMV proliferation during infection.

### 3.9. Dynamic Responses of the Tobacco Antioxidant System and Oxidative Damage Under Drought and TMV Stress

**Figure 10:**
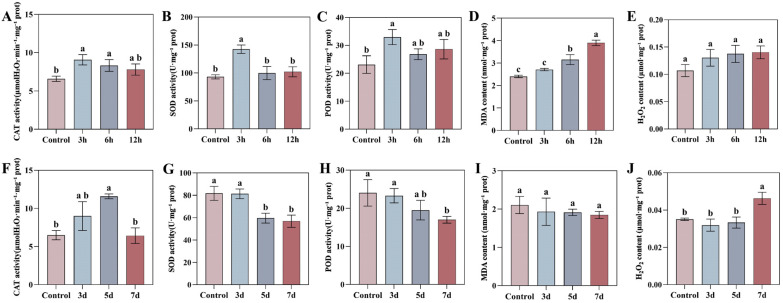
Temporal dynamics of oxidative stress markers and antioxidant enzymes in tobacco leaves under drought stress and TMV infection. (**A**–**E**) Drought stress: (**A**) CAT activity, (**B**) SOD activity, (**C**) POD activity, (**D**) MDA content, (**E**) H_2_O_2_ content. (**F**–**J**) TMV infection: (**F**) CAT activity, (**G**) SOD activity, (**H**) POD activity, (**I**) MDA content, (**J**) H_2_O_2_ content. Data are presented as mean ± SD (*n* = 3). Different lowercase letters indicate significant differences among time points (*p* < 0.05) determined by one-way ANOVA followed by Tukey’s HSD test.

To investigate the physiological dynamics of oxidative stress, we measured the activities of key antioxidant enzymes—superoxide dismutase (SOD), catalase (CAT), and peroxidase (POD)—and the levels of oxidative damage markers (malondialdehyde, MDA, and hydrogen peroxide, H_2_O_2_) in tobacco leaves under drought stress and Tobacco mosaic virus (TMV) infection over a time course. These physiological responses were then correlated with the expression patterns of *NtaFBXL* genes (Figure 10).

Drought stress triggered a rapid oxidative burst and a coordinated antioxidant defense response. The activities of CAT, SOD, and POD peaked simultaneously at 3 hpt, reaching 1.38-fold, 1.53-fold, and 1.43-fold of the control levels, respectively (*p* < 0.05), before gradually declining. The H_2_O_2_ content showed an increasing trend post-treatment, but the differences compared to the control did not reach statistical significance throughout the treatment period. While the MDA content, an indicator of membrane lipid peroxidation, began to accumulate significantly at 6 hpt and peaked at 12 hpt, indicating that prolonged drought ultimately caused substantial membrane damage. Notably, the peak activities of SOD, POD, and CAT at 3 hpt were temporally synchronized with the significant upregulation of several *NtaFBXL* genes (e.g., *NtaFBXL12*, *NtaFBXL35*) during the early stages of drought treatment (Figure 8). This temporal coincidence, combined with the established role of FBXL proteins as E3 ubiquitin ligases in stress signaling, suggests that these *NtaFBXL* genes may act as early response elements. They could potentially coordinate the initiation of rapid antioxidant defense under drought stress through the modification of specific signaling proteins. It is critical to note, however, that this correlative evidence does not demonstrate direct molecular interaction or regulatory control. Whether NtaFBXL proteins directly influence the activity or turnover of antioxidant enzymes remains to be determined by future studies.

In contrast to drought stress, TMV infection induced a gradual and generally suppressed oxidative stress response pattern. The activities of the core antioxidant enzymes, SOD and POD, showed a declining trend post-infection and decreased continuously over time. CAT activity exhibited a transient induction peak at 5 days post-inoculation (dpi) but returned to control levels by 7 dpi. Correspondingly, H_2_O_2_ content increased significantly in the late stage of infection (7 dpi), with an approximate 32% increase (*p* = 0.0045), whereas the MDA content showed no significant change throughout the infection process. This progressive suppression of antioxidant capacity, coupled with the late accumulation of H_2_O_2_, coincided with the significant downregulation of genes such as *NtaFBXL22* and *NtaFBXL42* (Figure 9). Given the significant negative correlation between the expression of these genes and TMV accumulation, we propose a plausible hypothesis: TMV may actively suppress the expression of these defense-associated host E3 ubiquitin ligase genes, thereby interfering with their mediated positive regulation of defense signaling or antioxidant proteins, weakening the host’s antioxidant capacity, and consequently facilitating viral proliferation.

## 4. Discussion

The *FBXL* family of F-box proteins represents a critical regulatory node within the ubiquitin–proteasome system, governing the targeted degradation of substrates to fine-tune plant growth and stress adaptation. While extensively characterized in model plants like *Arabidopsis*, our understanding of this family in the complex allotetraploid genome of tobacco (*Nicotiana tabacum* L.) has remained limited. This study provides the first comprehensive genome-wide analysis of the *NtaFBXL* family, revealing 47 members and delineating their roles in development, hormone signaling, and, most notably, in coordinating responses to abiotic and biotic stresses, including TMV infection.

Phylogenetic and synteny analyses uncovered significant lineage-specific expansion within Clade III of the *NtaFBXL* family (Figure 1 and Figure 4A). This expansion, primarily driven by segmental duplication events, is a recurring theme observed in the *FBXL* families of other polyploid plants, underscoring the pivotal role of whole-genome duplication (WGD) in the evolution of this gene family. For instance, a parallel expansion was reported in soybean (*Glycine max*), where a systemic analysis of the *GmFBXL* family revealed that the vast majority of its members (36 out of 45) originated from segmental duplication, with the evolutionary timeframe of this expansion (~16.24 MYA) coinciding with a known WGD event in soybean history [30]. Similarly, a parallel expansion driven by segmental duplications was also reported in upland cotton (*Gossypium hirsutum*) [31]. The strong purifying selection (Ka/Ks < 1) acting on duplicated *NtaFBXL* pairs (Figure 4C, Table S4), which is also a hallmark of the *GmFBXL* family (with 28 out of 29 duplicated pairs under purifying selection), indicates widespread evolutionary constraints to maintain essential protein functions across species [30,47,48,49]. The convergence of these evolutionary patterns-WGD-driven expansion coupled with strong purifying selection-in tobacco, soybean, and cotton suggests that the *FBXL* family is under stringent functional constraints.

The hypothesis that *NtaFBXL* genes are integral to stress signaling is strongly supported by the convergence of promoter architecture and expression dynamics. The abundance of stress- and hormone-responsive *cis*-elements, such as ABRE, MBS, and AuxRR-core (Figure 5), provides a mechanistic basis for their observed transcriptional regulation. Our qPCR data confirm this, showing that genes like *NtaFBXL2* and *NtaFBXL19* are co-induced by drought, ABA, and IAA (Figure 8). This coordinated upregulation suggests that these genes may function as hubs at the intersection of drought and hormone signaling cascades, a phenomenon crucial for orchestrating complex environmental responses [50,51,52].

GO enrichment analysis revealed that *NtaFBXL* genes were significantly enriched in the “floral organ morphogenesis” term (*p* = 5.57 × 10^−7^, Figure 6B). Furthermore, RNA-seq data indicated that the expression levels of these genes in flowers were higher than those in leaves (Figure 7)—a pattern consistent with the evolutionary conservation of *GmFBXL12* function in soybean, where *GmFBXL12* affects yield by regulating seed development [30]. Although tobacco has leaves as its primary economic organ, this result suggests that *NtaFBXL* may be involved in reproductive growth via the ubiquitination-mediated degradation of floral development regulators (e.g., SPL proteins) [53,54,55], providing a reference for yield regulation in Solanaceous crops such as tomato and pepper.

Under drought and TMV stress, dynamic changes in the activities of antioxidant enzymes and levels of oxidative damage markers in tobacco leaves showed a significant correlation with the expression timing of *NtaFBXL* genes, implying *NtaFBXL* may be involved in redox homeostasis regulation—this aligns with findings from the wheat F-box protein TaFBA1, which is transcriptionally upregulated by oxidative stress and enhances plant oxidative tolerance [56]. This correlation implies that *NtaFBXL* may be involved in the regulation of redox homeostasis; however, its role is only an indirect association—whether NtaFBXL proteins directly interact with or modify antioxidant enzymes or redox signaling components awaits future investigation. Verification of its direct impact on oxidative indices requires gene knockout/overexpression experiments, which represents one of the main limitations of this study.

The most innovative finding of this study is that the expression levels of six *NtaFBXL* genes (*NtaFBXL2/6/11/22/38/42*) showed a significant negative correlation with the accumulation of TMV coat protein (CP1). This finding suggests that TMV may impair host antiviral defense alongside the suppression of FBXL expression, which aligns well with the universal counter defensive strategies of plant viruses [57]. For example, during Alfalfa mosaic virus (AMV) infection in potato, the virus selectively suppresses the expression of defense-related genes in the host innate immune pathway (e.g., genes encoding PR proteins and MAP kinases), which represents a typical counter defensive tactic to evade host immunity [58]. Additionally, posttranscriptional gene silencing (PTGS)—a pivotal antiviral defense mechanism in plants—often has its key components targeted for suppression by viruses to promote infection [56]. From a molecular mechanism perspective, the suppression of *NtaFBXL* by TMV reflects the precise manipulation of the host ubiquitin–proteasome system (UPS) by viruses. During long-term coevolution between plant viruses and their hosts, ubiquitin ligases have become frequent targets of viral attack due to their central role in regulating immune signaling [59,60]. For instance, the P2 protein of Rice stripe virus (RSV) directly interacts with components of the host SCF ubiquitin ligase complex, disrupting F-box protein-driven ubiquitination and degradation processes [59,61]. More notably, the P0 protein of Polerovirus encodes an intrinsic F-box domain, which hijacks the host SCF complex to degrade AGO1 (a key effector of RNA silencing-mediated antiviral defense) [60]. As a functional F-box-containing subunit of the SCF complex, the suppression of *NtaFBXL* expression by TMV likely represents a conserved viral strategy to evade “ubiquitination-mediated degradation of viral proteins”. This observation is consistent with the conclusion by Zhang et al. (2025) that “evolutionarily distinct viral proteins tend to target core host immune components” [59]. Collectively, our findings provide the first evidence directly linking *NtaFBXL* genes to TMV stress, suggesting that the virus may weaken the host’s defense capacity by suppressing the expression of these host E3 ubiquitin ligase genes—a potential mechanism in plant–virus interactions that has not yet been fully explored. Given that TMV poses a major threat to tobacco leaf yield and quality, understanding how the *FBXL* gene family responds to viral infection could provide novel molecular targets for developing virus-resistant varieties [62,63].

While this study establishes a strong correlation between *NtaFBXL* expression and various stress responses, it is important to acknowledge its primary limitation: the correlative nature of the evidence and the lack of direct functional validation. Our conclusions are built upon in silico predictions, transcriptomic analyses, and correlative physiological data, which together generate robust and valuable hypotheses. However, the specific protein substrates of these *NtaFBXLs* and their precise mechanistic roles in stress signaling pathways remain unknown. Therefore, the most immediate future direction involves functional characterization, such as knocking out or overexpressing the TMV-correlated genes to validate their role in viral resistance and to identify their ubiquitination targets through interactome studies. Such efforts will be crucial to move from correlation to causation and fully exploit the potential of *NtaFBXL* genes for engineering stress-resilient crops.

## 5. Conclusions

This study presents a comprehensive genomic and functional analysis of the *FBXL* gene family in tobacco (*Nicotiana tabacum* L.). A total of 47 *NtaFBXL* genes were identified, with phylogenetic and synteny analyses revealing a notable expansion in Clade III, an evolutionary pattern consistent with the polyploid history of the species. Promoter analysis predicted an abundance of stress- and hormone-related cis-elements, which was corroborated by expression data showing that *NtaFBXL* genes are responsive to drought, ABA, and IAA treatments. Furthermore, a significant negative correlation was observed between the expression of six *NtaFBXL* genes (*NtaFBXL2/6/11/22/38/42*) and the accumulation of Tobacco Mosaic Virus (TMV). Temporal changes in antioxidant enzyme activities and oxidative markers under drought and TMV stress were also documented, occurring in parallel with the expression dynamics of specific *NtaFBXL* genes.

Based on these correlative datasets, this study generates several testable hypotheses that provide a framework for future functional research. We propose that specific *NtaFBXLs* whose expression is suppressed during TMV infection are candidate host factors potentially involved in antiviral defense, and that the coordinated expression patterns between certain *NtaFBXLs* and the antioxidant system suggest a putative (though likely indirect) role for this family in modulating redox homeostasis under stress. The precise molecular functions, specific protein substrates, and causal roles of *NtaFBXLs* in these stress responses remain to be determined. Future work employing genetic manipulation, protein interaction assays, and ubiquitination target identification will be essential to validate these hypotheses and exploit the potential of the *NtaFBXL* family for improving stress resilience in crops.

## Data Availability

All datasets generated or analyzed during this study are included in this published article and its supplementary information files, and the publicly available datasets (RNA-seq data) analyzed during this study can be found in the NCBI SRA under accession PRJNA208209. Further inquiries can be directed to the corresponding author.

## Supplementary Materials

The following supporting information can be downloaded at: https://www.mdpi.com/article/10.3390/antiox15020246/s1, Table S1: Primer sequences of qPCR; Table S2: Basic information of the *NtaFBXL* gene family; Table S3: Syntenic relationships among *FBXL* genes. Table S4: Ka/Ks analysis and evolutionary parameters of collinear *NtaFBXL* gene pairs in *Nicotiana tabacum*.
